# EFFECTS OF RESISTANCE EXERCISE TRAINING ON ENDOTHELIAL FUNCTION AND MUSCLE PERFUSION IN OLDER ADULTS WITH DIABETES

**DOI:** 10.1093/geroni/igz038.3454

**Published:** 2019-11-08

**Authors:** Amanda Randolph, Tatiana Moro, Adetutu Odejimi, Blake Rasmussen, Elena Volpi

**Affiliations:** 1 UNIVERSITY OF TEXAS MEDICAL BRANCH, Galveston, Texas, United States; 2 Sealy Center on Agyng, Galveston, Texas, United States; 3 UTMB, Galveston, Texas, United States

## Abstract

BACKGROUND: Sarcopenia contributes to frailty, disability, and dependence in older adults, and is accelerated by Type 2 Diabetes Mellitus (T2DM). In addition to its direct role in increasing muscle mass, progressive resistance exercise training (PRET) may also reduce sarcopenia by improving endothelial function and muscle perfusion. METHODS: Fifteen older adults with uncomplicated and well-controlled T2DM participated in a PRET program 3 times weekly for 3 months. Prior to and immediately following the intervention, flow-mediated dilation testing was performed to assess large vessel endothelial function via ultrasound and muscle perfusion via near-infrared spectroscopy (NIRS). RESULTS: Preliminary ultrasound data from 9 subjects show a significant increase (5.21% to 8.73%, p=0.0448) in percent flow mediated dilation (%FMD), suggesting a modest improvement in endothelial function after 3 months’ PRET. Preliminary NIRS data from 7 subjects showed no significant changes in oxygen saturation or reperfusion rates as a result of the intervention. CONCLUSION: Our preliminary data indicate that, in older adults with T2DM, 3 months’ PRET is associated with modestly improved endothelial function in large vessels (as demonstrated by a significant increase in %FMD), but does not appear to be associated with improvements in muscle perfusion

